# A systematic review for the development of Alzheimer’s disease in *in vitro* models: a focus on different inducing agents

**DOI:** 10.3389/fnagi.2023.1296919

**Published:** 2023-12-20

**Authors:** Manisha Prajapat, Gurjeet Kaur, Gajendra Choudhary, Paras Pahwa, Seema Bansal, Rupa Joshi, Gitika Batra, Abhishek Mishra, Rubal Singla, Harminder Kaur, Praisy K. Prabha, Ajay Prakash Patel, Bikash Medhi

**Affiliations:** ^1^Department of Pharmacology, PGIMER, Chandigarh, India; ^2^MM College of Pharmacy, Maharishi Markandeshwar (DU) University, Mullana, Ambala, India; ^3^Department of Neurology, PGIMER, Chandigarh, India; ^4^Department of Biomedical Sciences, University of Minnesota, Minneapolis, MN, United States

**Keywords:** Alzheimer’s disease, *in-vitro* models, neurodegeneration, tau protein, amyloid beta

## Abstract

Alzheimer’s disease (AD) is the most common progressive neurodegenerative disease and is associated with dementia. Presently, various chemical and environmental agents are used to induce *in-vitro* models of Alzheimer disease to investigate the efficacy of different therapeutic drugs. We screened literature from databases such as PubMed, ScienceDirect, and Google scholar, emphasizing the diverse targeting mechanisms of neuro degeneration explored in *in-vitro* models. The results revealed studies in which different types of chemicals and environmental agents were used for *in-vitro* development of Alzheimer-targeting mechanisms of neurodegeneration. Studies using chemically induced *in-vitro* AD models included in this systematic review will contribute to a deeper understanding of AD. However, none of these models can reproduce all the characteristics of disease progression seen in the majority of Alzheimer’s disease subtypes. Additional modifications would be required to replicate the complex conditions of human AD in an exact manner. *In-vitro* models of Alzheimer’s disease developed using chemicals and environmental agents are instrumental in providing insights into the disease’s pathophysiology; therefore, chemical-induced *in-vitro* AD models will continue to play vital role in future AD research. This systematic screening revealed the pivotal role of chemical-induced *in-vitro* AD models in advancing our understanding of AD pathophysiology and is therefore important to understand the potential of these chemicals in AD pathogenesis.

## 1 Introduction

Alzheimer’s disease (AD) is a neurodegenerative disease that is considered the most common cause of dementia. The key pathological hallmarks of AD are plaque and tangle formation arising due to amyloid beta deposition and hyper phosphorylation of tau in brain tissues, respectively (Basaly et al., 2021; Grant et al., 2021). The main clinical symptoms of Alzheimer’s disease include cognitive dysfunction and progressive loss of memory. There are multiple factors responsible for AD, such as genetic mutations, epigenetic factors, aging, and external environmental factors, leading to abnormal neuronal function (Tiwari et al., 2019; Basaly et al., 2021). The German neurologist Dr. Alois Alzheimer discovered this neurodegenerative disease in 1906 while treating a 51-year-old patient who was suffering from memory loss, language problems, hallucinations, and disorientation (Tiwari et al., 2019; Reitz et al., 2020). AD is a major contributor to the overall global epidemic, with approximately 48.6 million people suffering from AD or dementia worldwide. Approximately 10% of people 65 years of age or above are affected by AD (Wang et al., 2021). It is expected that the cases of AD will rise at a highly accelerated rate by 2050. To date, there is no proper cure or prevention for Alzheimer’s disease. Furthermore, the exact mechanism of AD pathogenesis is still elusive. According to previous studies, the pathological characteristics of AD are defined by the extracellular accumulation of insoluble amyloid beta protein, intracellular aggregation of hyperphosphorylated tau protein, mitochondrial dysfunction, increased inflammation, and oxidative stress–induced apoptotic activity (McCarthy et al., 2004; Gan et al., 2014; Rai et al., 2020b). Many environmental factors and chemicals have been identified in the past for inducing AD models in different *in vitro* and *in vivo* studies (Wang B. R. et al., 2018). Furthermore, the increasing prevalences of various metabolic diseases such as diabetes have also been linked to neurodegeneration as well the pathogenesis of AD (Martins, 2018).

To date, there is no systematic review study that has thoroughly investigated the chemical agents that can be used to induce *in vitro* AD models. Thus, this is the first study to compile all the AD inducible agents in *in-vitro* models. The first objective of this study is to determine the number of chemical and environmental agents that have been utilized to induce Alzheimer’s disease (AD) in *in vitro* models up to the present time. The second objective is to identify those agents that have demonstrated the potential to induce pathological features of AD, such as amyloid beta accumulation and tau hyperphosphorylation, in cell culture models. Thirdly, we seek to uncover previously unknown *in-vitro* agents that can be employed to establish a reliable and replicable AD *in-vitro* model.

To achieve these three objectives, we conducted a systematic review of all original research studies focused on Alzheimer’s disease induced in *in vitro* models. Therefore, in this study, we systematically reviewed all *in-vitro* studies involving AD models to enlist different types of AD inducing agents. Through this study, we compared various AD-inducing agents and also explored the underlying pathological pathways associated with the disease.

## 2 Materials and methods

### 2.1 Search strategy

The search strategy was based on the PICO (Population, Intervention, Control, and Outcomes) format, with the approach as follows: P—*In vitro* inducible AD model; I—None; C—None; and O—Presence of amyloid beta accumulation/tau hyperphosphorylation/neuronal toxicity. Four authors independently searched the selected databases for inclusion of relevant studies in the systematic review.

### 2.2 Database screening

**Figure 1:**
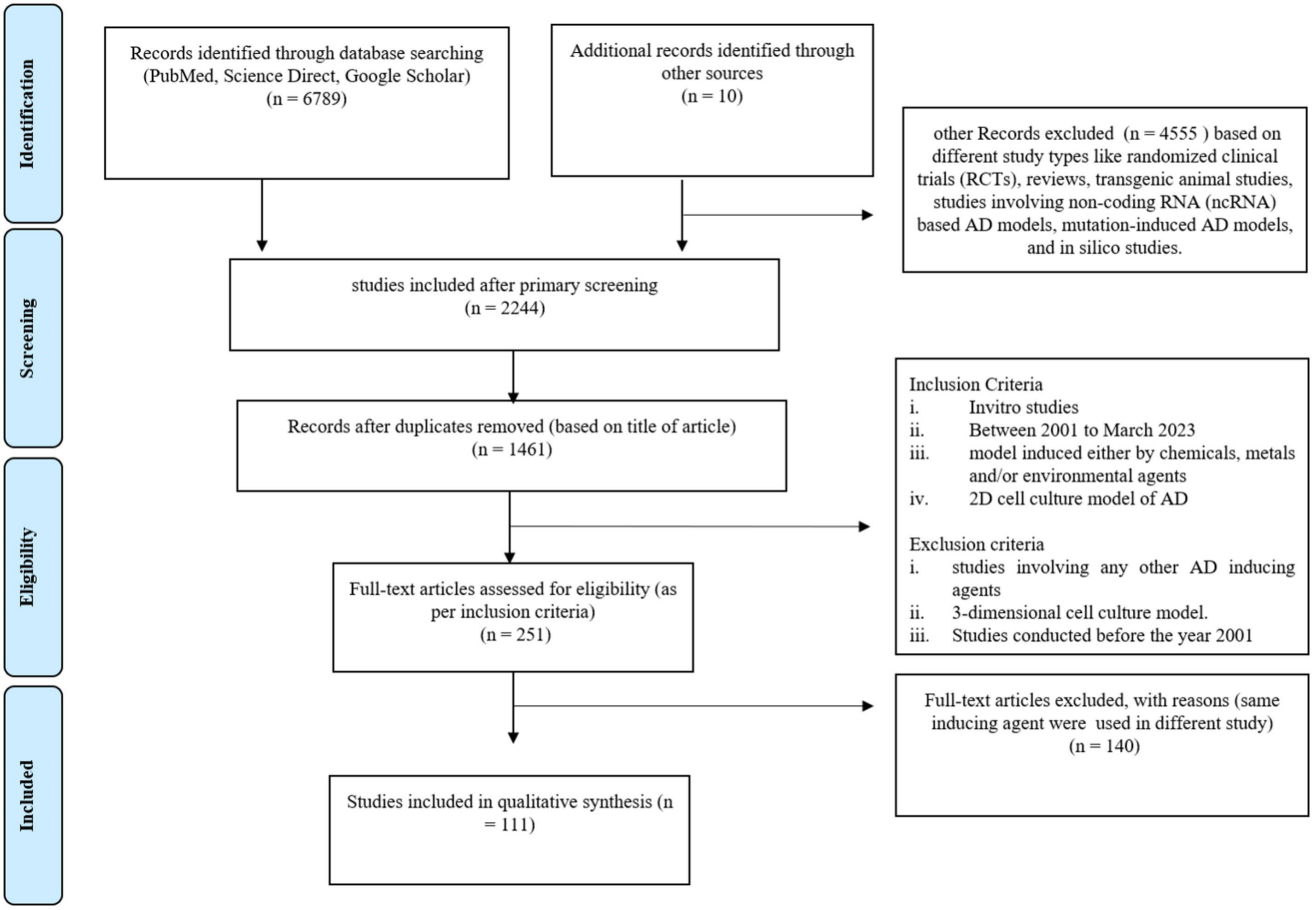
Prisma chart of this systematic review study.

We screened PubMed, Science Direct, and Google Scholar for the keywords “chemically induced Alzheimer disease model *in vitro* study,” “Alzheimer disease” AND “cell culture,” “inducing agent” AND “Alzheimer disease,” and “Alzheimer disease” AND “*in vitro*,” from the year 2001 to 24 March 2023. The search result files were extracted and initially screened based on the title and abstract using Rayyan QCRI. Further screening of full text articles was carried out to identify studies for inclusion in the systematic review. Research articles involving the use of an induced *in vitro* AD model were included for further reviewing. The studies were screened as per the criteria described in the Prisma chart (Figure 1). The basis of inclusion was primarily the identification of different chemicals, metals, and environmental agents that were being used for *in vitro* studies.

## 3 Results

A total of 6,799 articles were extracted after preliminary screening of the databases, out of which, 98 duplicate studies were removed. In the initial screening, 4,555 articles were excluded on the basis of a different study type, such as randomized clinical trials (RCTs), reviews, transgenic animal studies, studies involving non-coding RNA (ncRNA)–based AD models, mutation-induced AD models, and *in silico* studies. The primary screening results revealed 2,244 studies. Further, based on the title of the article, 1,461 studies were included and a total of 484 articles were excluded. Further, 1,210 articles were excluded based on different study types, such as randomized clinical trials (RCTs), reviews, transgenic animal studies, studies involving non-coding RNA (ncRNA)–based AD models, mutation-induced AD models, and *in silico* studies. The remaining 251 articles were screened for inclusion of *in vitro* and *in vivo* studies with only chemical- and environmental-based agents to induce the AD model. Based on the use of different inducing agents in *in-vitro* studies, we selected 111 articles for this systematic review. These articles were only related to original research including *in-vitro* models of AD research. The details of the included studies, such as the inducing agents used and the molecular markers studied for expression are summarized in Table 1.

**Table 1:** Details of different studies on inducing agent and their molecular marker expression.

S. No.	Title	Chemical	Neurotoxic mechanism	*in vivo/in vitro*	Cell line	Reference
	Aluminium chloride hexahydrate and Maltol toxicity decreased by using Asiatic acid in *in vitro* model of AD	Aluminium chloride hexahydrate and Maltol	Down regulates PI3K/AKT/GSK-3β pathway	*In vitro*	SH-SY 5Y cells	Mashoque et al. (2018)
	PM2.5 exposure worsens oligomeric amyloid beta-induced neuronal damage and enhances NLRP3 inflammasome activation in an Alzheimer’s disease *in vitro* model.	PM2.5	IL-1 production and NLRP3 inflammasome activation are both increased.	*In vitro*	Primary microglial cells	Wang B. R. et al. (2018)
	In an *in vitro* and *in vivo* model of Alzheimer’s disease hydroxyurea affects membrane fluidity which further affects neuronal membrane aging	Hydroxyurea	Increase inAβ levels and decrease in AMPK/ACC/CPT1 pathway	*In Vivo* and *in vitro*	Rat primary cortical neurons	Yu and Cheng (2021)
	Neuroinflammation in rats caused by inducing AlCl_3_ and checking the effect of fusion of Curcumin and Resveratrol against it. Caffeic acid’s effect on aluminium chloride-induced dementia in rats.	AlCl_3_	High levels of neuro-inflammation as well as increase in COX-2 expression inhibitor High levels of AChE	*In vitro* and *In vivo*	PC-12 cells Rat brain homogenate	Mentis et al. (2021) and Sanajou et al. (2023)
5.	In SH-SY5Y and C6 cells treated with 27-hydroxycholesterol, NF-B-mediated inflammatory damage is altered differently.	27-Hydroxycholesterol	[In SH-SY5Y] TNF and iNOS secretion increased, while IL10 secretion decreased; TGF, NFB p65 and p50 expression increased, while COX2 expression decreased. [In C6 cells] TLR4 and TGF expression increased, while IL1, IL10, TNF, and iNOS secretion decreased.	*In vitro*	SH-SY5Y and C6 cells	Ma et al. (2019)
6.	Rosiglitazone Embedded Nanocarrier System Has Neuroprotective Potential in Streptozotocin-Induced Alzheimer’s Disease Mice. In diabetics, sarsasapogenin reduces Alzheimer’s-like encephalopathy. The interaction of hyperphosphorylated tau and pyroptosis in forskolin and streptozotocin-induced Alzheimer’s disease models.	Streptozotocin	TNF- (tumor necrosis factor) and interleukin-6 levels rise (IL-6). Increase in AKT/GSK-3β cascade High levels of hyperphosphorylated tau and increased expression of NLRP1 and caspase-1	*In vitro* and *in vivo*	SH-SY5Y cells SH-SY5Y cells PC12 cells	Sarathlal et al. (2021), Tripathi et al. (2019), and Hampel et al. (2018)
7.	The Dephosphorylation of p70S6 (Thr389) Kinase as a Marker of L-Glutamate-Induced Excitotoxicity Related to Diabetes Disturbances—an Unusual *In Vitro* Mode	L-Glutamate	Increased phosphorylation of p70S6K in Thr^389^ residue	*In vitro*	PC12 cells	Rorbach-Dolata et al. (2020)
8.	The ameliorative potential of desalted *Salicornia europaea* L. extract in a multifaceted Alzheimer’s-like scopolamine-induced amnesic mouse model. Tert-butyl-4-hydroxy-3-((3-(2-methylpiperidin-yl) propylcarbamoyl)phenyl)carbamoylcarbamoylcarbamoylcarbamoylcarbamoylcarbamoylcarbamoylcarbamoylcarb In Astrocytes Stimulated with Amyloid Beta 1–42 and in a Scopolamine Model, carbamate Has Moderate Protective Activity. Lavender oil protects mice from scopolamine-induced cognitive deficits and PC12 cells from H2O2-induced cytotoxicity. Pretreatment of Sulforaphane-Enriched Broccoli Sprouts with Pulsed Electric Fields Its antioxidative ability, mediated by Nrf2-HO-1 activation, reduces neuroinflammation and improves scopolamine-induced amnesia in the mouse brain. *Schisandra chinensis* and *Ribes fasciculatum* Work Together to Prevent Neuronal Cell Death and Scopolamine-Induced Cognitive Impairment in Rats. Myricetin improves scopolamine-induced memory impairment in mice by inhibiting acetylcholinesterase and decreasing brain iron levels.	Scopolamine	TNF-, IL-1, and IL-6 mRNA levels all increased. Increase in (TNFα), (IL-1β), (IL-6), and the overexpression of (GFAP) Increase in LDH, ROS and NO levels. Decrease in MMP levels Activates NF-κB signaling Increased expression of Nrf2 and HO-1 Increase in AChE activity and decreasing MDA level	*In vivo* and *in vitro*	BV-2 cells Astrocyte cells PC12 cells BV2 cells PC 12 cells SH-SY5Y cells	Karthivashan et al. (2018), Yang et al. (2021), Rai et al. (2021b), Bhatti et al. (2022), Kawahara et al. (2001), and Tuneva et al. (2006)
9.	*In vitro* and *in vivo*, curcumin and hesperetinreduce D-galactose-induced brain senescence.	D-galactose	Up-regulate expression of p16 and p21 and lower expression of SOD1, Gpx1, and catalase	*In vitro*	SH-SY5Y cells	Lee et al. (2020)
	In aged rats, hispidulin prevents sevoflurane-induced memory dysfunction.	sevoflurane	Increase inAβ accumulation and neuroinflammation. High level of NF-κB	*In vivo* and *in vitro*	H4 cells	Huang et al. (2018)
	*In vivo* and *in vitro*, ginsenoside Rd. protects against okadaic acid-induced neurotoxicity. Using Mesenchymal Stem Cell-Conditioned Medium, Extracellular Vesicle Mitochondrial Transfer Improves Mitochondrial Dysfunction and Suppresses Apoptosis in Okadaic Acid-Treated SH-SY5Y Cells.	Okadaic acid	High level of protein phosphatase 2A. Increases p181-tau expression	*In vitro* and *in vivo* *In vitro*	Cortical neurons cell culture SH-SY5Y Cells	Li et al. (2011)
	Manganese-induced cognitive impairment is caused by dysregulated APP expression and -secretase processing of APP.	MnCl_2_.4H_2_O	APP, −secretase, and soluble APP alpha protein (sAPP) expression were all inhibited.	*In vivo* and *in vitro*	Neuro-2a (N2a) cells	Gu et al. (2018)
	*In vitro*, aluminium causes tau aggregation, but not *in vivo*.	Al-maltolate	Induces Tau aggregation	*In vitro*	Neuronal cell line (N2a)	Mizoroki et al. (2007)
	Investigating the molecular mechanisms of neurodegenerative diseases: Differential protein expression in hippocampal cells linked to heavy metal (Pb, As, and MeHg) neurotoxicity.	Lead chloride, Sodium metaarsenite, Methyl mercury chloride	Depletion of ATP production and Alteration of proteins in complex I–V, Increase in ROS levels	*In vitro*	HT-22 cells	Karri et al. (2018)
	Aluminum Modifies Amyloid1–42 Effects on Neuronal Calcium Homeostasis and Mitochondrial Function in a Triple Transgenic Alzheimer’s Disease Mouse Model.	Aluminum	Promotes Aβ aggregation High levels of Ca^++^ ions	*In vitro*	Cortical neuronal cultures	Drago et al. (2008)
	Tranylcypromine, an MAO inhibitor, modifies LPS- and A-mediated neuroinflammatory responses in wild-type mice and an Alzheimer’s disease mouse model. Piperlongumine Suppresses the NF-KappaB Pathway, which improves Lipopolysaccharide-Induced Amyloidogenesis. Deoxyelephantopin improves memory impairments caused by lipopolysaccharides (LPS) in rats: evidence for anti-neuroinflammatory properties. Effects of 4-O-methylhonokiol on lipopolysaccharide-induced neuroinflammation, amyloidogenesis, and memory impairment *in vitro* and *in vivo* via inhibition of nuclear factor-kappaB. Astaxanthin Reduces Lipopolysaccharide-Induced Neuroinflammation, Oxidative Stress, and Memory Dysfunction by Inactivating the Signal Transducer and Activator of Transcription 3 Pathway.	Lipopolysaccharide	Increases IL-1β and IL-6 mRNA levels Activates NF-κB pathway, It increases the level of neuroinflammation markers (COX-2 and iNOS) High levels of COX-2 and iNOS expression Increased expressions of cytosolic group IV phospholipase A2, 5-lipoxygenase and toll-like receptor-4 as well as high level of ROS generation And Neuroinflammation High levels of TNF-α, IL-1β and IL-6	*In vitro*	BV2 microglial cells BV-2, and primary astrocyte cells BV-2 microglial cells BV-2 cells BV-2 microglial cells	Park et al. (2020), Gong et al. (2011), Andy et al. (2018), Lee et al. (2012), and Han et al. (2019)
	Formaldehyde induces hyperphosphorylation and polymerization of Tau protein both *in vitro* and *in vivo*	Formaldehyde	Increase hyperphosphorylation of tau protein	*in vitro and in vivo*	neuroblastoma (N2a)	Lu et al. (2013)

### 3.1 Pathophysiology of AD

There are several pathological pathways that are directly or indirectly involved in the neurodegeneration process of Alzheimer’ disease.

The most widely accepted hypothesis for AD pathogenesis is based on the cholinergic pathway, amyloid beta cascade, and tau hyperphosphorylation. These three hypotheses are linked by various interconnected pathway mechanisms.

According to the cholinergic hypothesis, the dysfunction of acetylcholine neurotransmission in neurons is majorly due to a reduction in acetylcholine-transferase enzyme, a reduction of acetylcholine receptors, or reduction of acetylcholine itself. Moreover, cholinergic loss is based on the degeneration of cholinergic neurons and their projecting axons to the cerebral cortex. The main components for acetylcholine synthesis are choline, acetyl coenzyme, and choline acetyltransferase (ChAT). Normally, acetylcholine is released from presynaptic neurons into the synapse, from where it further binds to postsynaptic receptors (M1) that subsequently transmit the signal to connecting neurons. Another enzyme, acetyl cholinesterase, is also responsible for the breakdown of acetylcholine into choline and acetate in the synaptic cleft, which are taken by cells for the process of recycling (Srivastava et al., 2019). Therefore, the deregulation of the enzymatic activity of acetylcholinesterase may also lead to AD (Hampel et al., 2018; Gothwal et al., 2019; Tripathi et al., 2019). In the case of the amyloid beta cascade hypothesis, Aβ is formed when the APP protein is digested by alpha, beta, and gamma secretase. Previous studies have shown that in the case of the non-amyloidogenic pathway, alpha and gamma-secretase enzymes produce a C-terminal fragment, soluble ectodomain, and cleave the APP transmembrane protein. Another pathway is the pathological amyloidogenic pathway, in which the APP protein is cleaved by beta and gamma-secretase enzymes, producing amyloid beta peptide fragments that are more prone to aggregation, thus leading to the development of insoluble oligomers and amyloid plaques. The accumulation of the insoluble oligomers induce synaptic dysfunction, inflammation, mitochondrial damage, hyperphosphorylation, and dysregulation of calcium homeostasis, ultimately leading to neuronal toxicity and degeneration (Esmieu et al., 2019; Fan et al., 2020; Rai et al., 2020a). Tau is a protein expressed by the MAPT (microtubule associated tau) gene and is found in the neuronal axons of the brain. The tau protein has a main roles in the maintenance of microtubule structure, transport function, and synapse structure. The phosphorylation and dephosphorylation status of the tau protein is dependent on the activity of protein kinase and protein phosphatase. In disease conditions, hyperphosphorylation of the tau protein results in structural alteration, leading to its detachment from microtubules, further resulting in a loss of microtubule stability and polymerization capacity (Ismail et al., 2017). This results in elevated tau protein levels in the cytoplasm, leading to tau aggregation and the formation of insoluble paired helical filaments (PHFs). The deposition of PHFs in the form of fibrillar neurofibrillary tangles (NFTs) causes neurotoxicity, cell dysfunction, and loss of synapses (Fan et al., 2020; Rai et al., 2020b).

Other routes underlying the etiology of AD, such as excitatory glutamatergic neurotransmission through N-methyl-d-aspartate receptor (NMDAR), result in triggered neuro excitotoxicity (Wang and Reddy, 2017). Dysregulation of Wnt signaling (wingless/integrated), the AKT pathway, MAPK/ERK pathway, JNK pathway (Jun N-terminal kinase), RAGE (receptor for advanced glycation end products) pathway, and AMPK (AMP-activated protein kinase) pathway have also been reported in previous studies and have been linked to the pathogenesis of Alzheimer’s disease (Atzori et al., 2001; Greco et al., 2009; Exil et al., 2014; Loera-Valencia et al., 2021; Ramakrishna et al., 2023).

Other pathways, such as activation of glial cells (like microglia and astrocytes), can induce oxidative and inflammatory reactions, which lead to neuronal dysfunction and apoptosis, and eventually Alzheimer’s disease. The microglial cells have a role in the clearance of amyloid plaque; however, aberrant gene expression of ABCA7 (ATP-binding cassette sub-family A member 7), CD3 (cluster of differentiation-3), CR1 (complement receptor type 1), SPI1, and TREM2c (triggering receptors expressed on myeloid cells 2c) can expedite the neuronal degeneration process. The disrupted phagocytic activity of microglia cells enhances the accumulation of amyloid beta deposition, subsequently leading to neuronal degeneration (McCarthy et al., 2004; Wang et al., 2021). Several studies have also reported the role of the transcription factor AP1 (activator protein 1), NFkB (nuclear factor k beta), SP 1, NRF2 (nuclear factor-erythroid factor 2), and TFEB (transcription factor EB) in initiating the process of neuro degeneration that might lead to the development of AD (Rai et al., 2021b).

**Figure 2:**
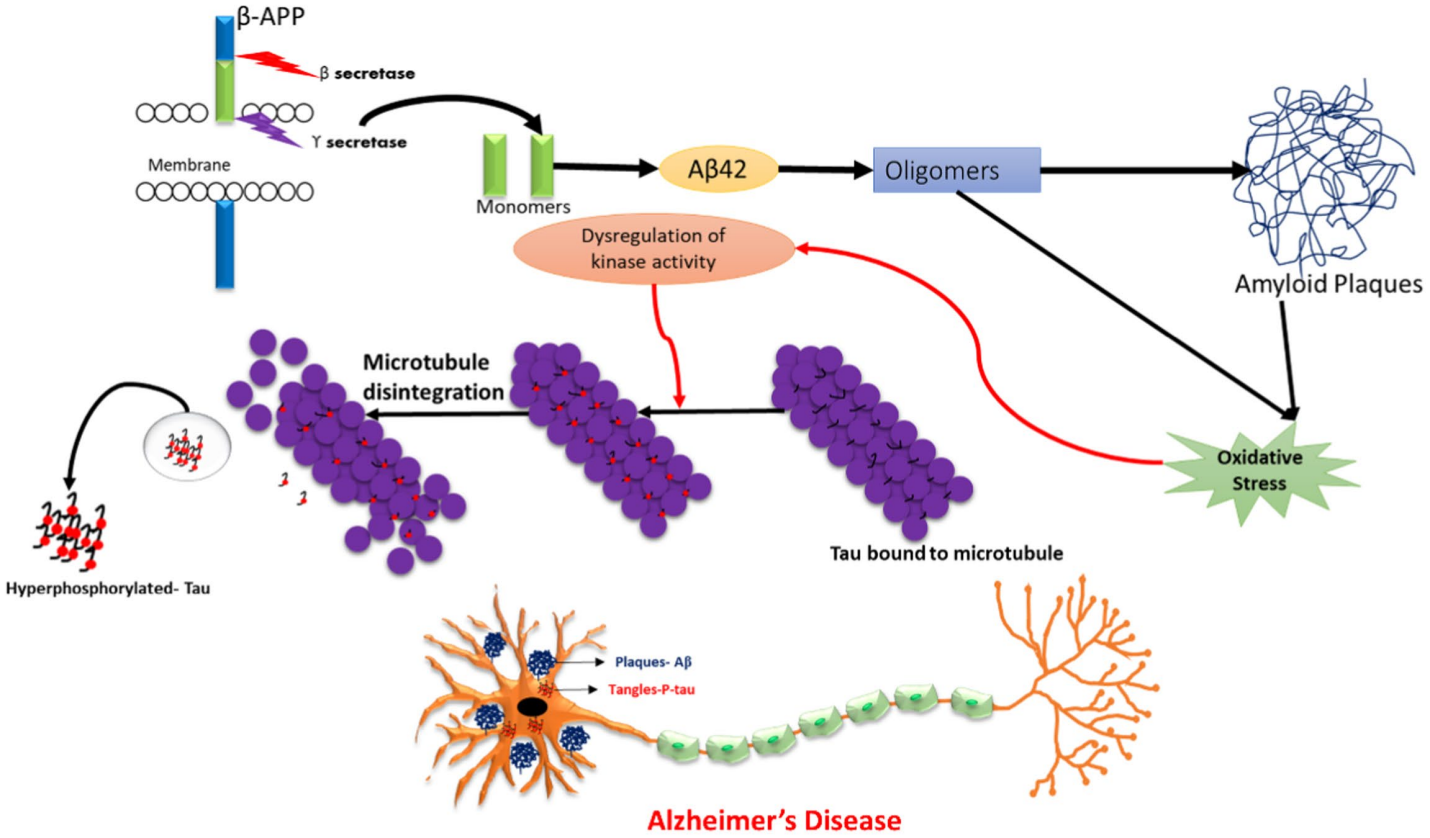
Schematic representation of Alzheimer’s Disease (AD) pathology.

The emerging world faces a genetic risk of Alzheimer’s disease (AD) due to mutations in PSEN1 (located on chromosome 14), PSEN2 (located on chromosome 1), and APP (located on chromosome 21), which are known to cause familial Alzheimer’s disease (FAD). Some non-genetic factors have also been identified to be involved in AD pathogenesis, such as smoking, hypercholesterolemia, obesity, vitamin deficiency, diabetes, hypertension, head trauma, stroke, and depression. The severity of metabolic diseases and traumatic injuries was shown to increase as a result of exposure to pesticides, an electromagnetic field, aluminium, lead, iron, copper, zinc, mercury, particulate matter, and solvents, which, directly or indirectly, might pose a risk for developing AD (Rahman et al., 2020; Rai et al., 2021a). Hence, all these environmental and chemical factors were able to induce plaque and tangle formation, increase oxidative stress, and decrease neprilysin (amyloid beta degrading enzyme), thereby increasing neuronal death. Thus, pre- and post-natal exposure to these environmental factors may promote the development of this neurodegenerative disease in later life (Chin-Chan et al., 2015; Esmieu et al., 2019). Nonetheless, numerous protective factors, including social contact, mental activity, nonsteroidal anti-inflammatory (NSAIDs) usage, coffee intake, moderate alcohol use, physical activity, and past vaccinations may lessen the chance of developing AD (Mentis et al., 2021). The pathophysiology of AD is represented in Figure 2.

## 4 Different agents used for induction of Alzheimer disease

The toxic effects of many agents, such as environmental, chemical, and xenobiotic agents, have been identified in previous *in vitro* and *in vivo* studies (Lu et al., 2013; Martins, 2018). Longer exposure to these toxic agents can be hazardous as they induce neuroinflammation and neuropathology, opening the door for AD development (Wang B. R. et al., 2018).

### 4.1 Agent targeted expression

**Figure 3:**
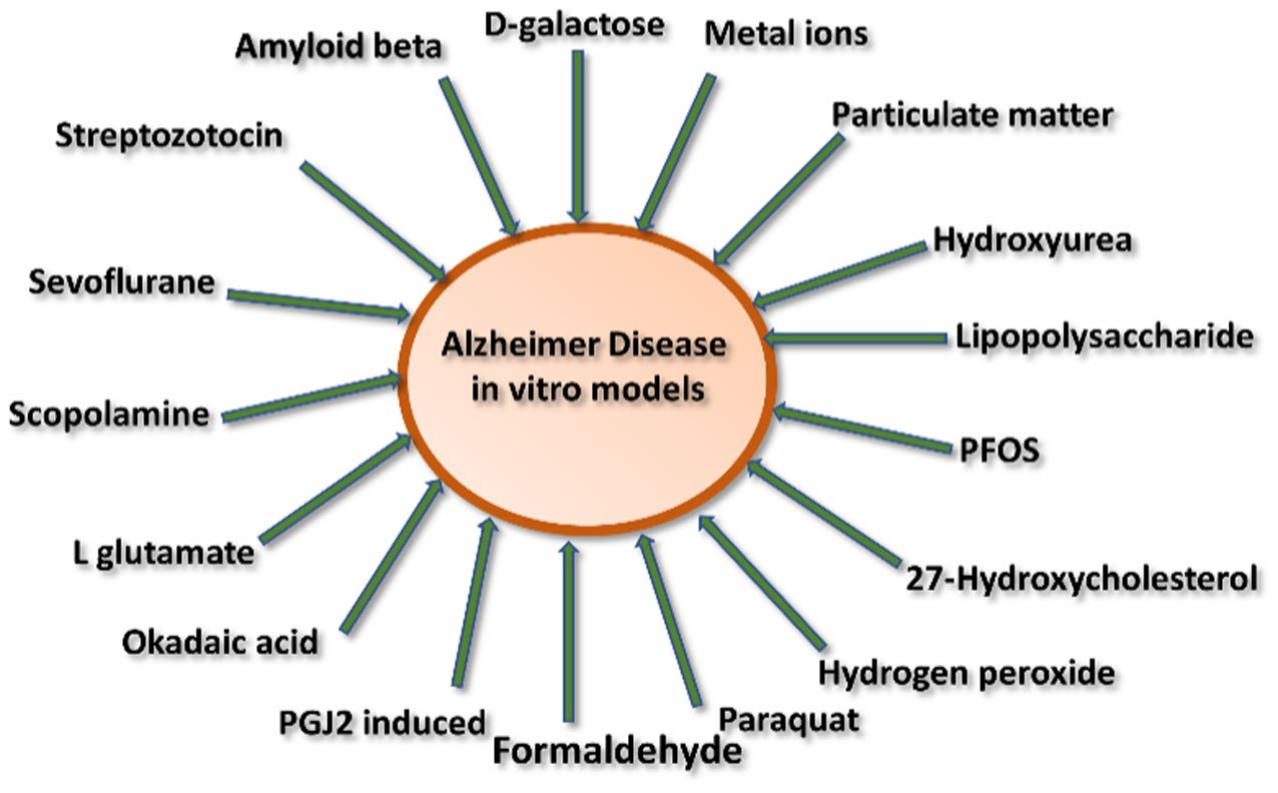
Schematic representation of various inducing agents involved in Alzheimer’s disease.

AD inducing agents can be divided into two categories: (1) environmental inducing agents and (2) chemically inducing agents (Figure 3).

### 4.2 Environmental inducing agents

Aluminum chloride hexahydrate (AlCl_3_H_12_O_6_), maltol, PM2.5 (particulate matter 2.5), hydroxyurea, AlCl_3_, 27-hydroxycholesterol, H_2_O_2_, MnCl_2_._4_H_2_O, Al-maltolate, lead, arsenic, and mercury.

### 4.3 Metals ion-induced *in vitro* AD models

Excessive exposure to heavy metals has been found to be involved in neurotoxicity and neuronal damage.

#### 4.3.1 Aluminum chloride

Aluminum is abundantly present as an environmental toxicant and is associated with neuropathological disease. Aluminum chloride enters the body via soil, food, pharmaceutical agents, and water, from where it may further accumulate in the brain and affect neurotransmitter synthesis, axonal transport, disturb the balance of protein phosphorylation and dephosphorylation, protein degradation, and gene expression, thereby inducing inflammation, apoptotic activity, and amyloid beta deposition. Al can also cause mitochondrial and endoplasmic reticulum dysfunction, leading to an increase in oxidative stress and apoptosis (Kawahara et al., 1994; Mustafa Rizvi et al., 2014). Therefore, aluminum is a possible inducing agent to cause neurotoxicity as well as neurodegenerative diseases such as Alzheimer’s disease (Tiwari et al., 2019).

In the mechanistic pathway, Al is able to decrease the nucleus translocation of NRF2, either directly or by indirectly activating GSK3 beta. Increased NRF2 levels in the cytoplasm phosphorylate the BAX apoptotic protein, which is further involved in the release of cytochrome-c from the mitochondria, leading to apoptosis activity. On the other hand, Al also activates GSK3 beta by inhibiting AKT and Wnt signaling pathways, which are further responsible for hyperphosphorylation of the tau protein, leading to synaptoxicity (Kawahara et al., 1994; Hong and An, 2018; Sanajou et al., 2023).

**Figure 4:**
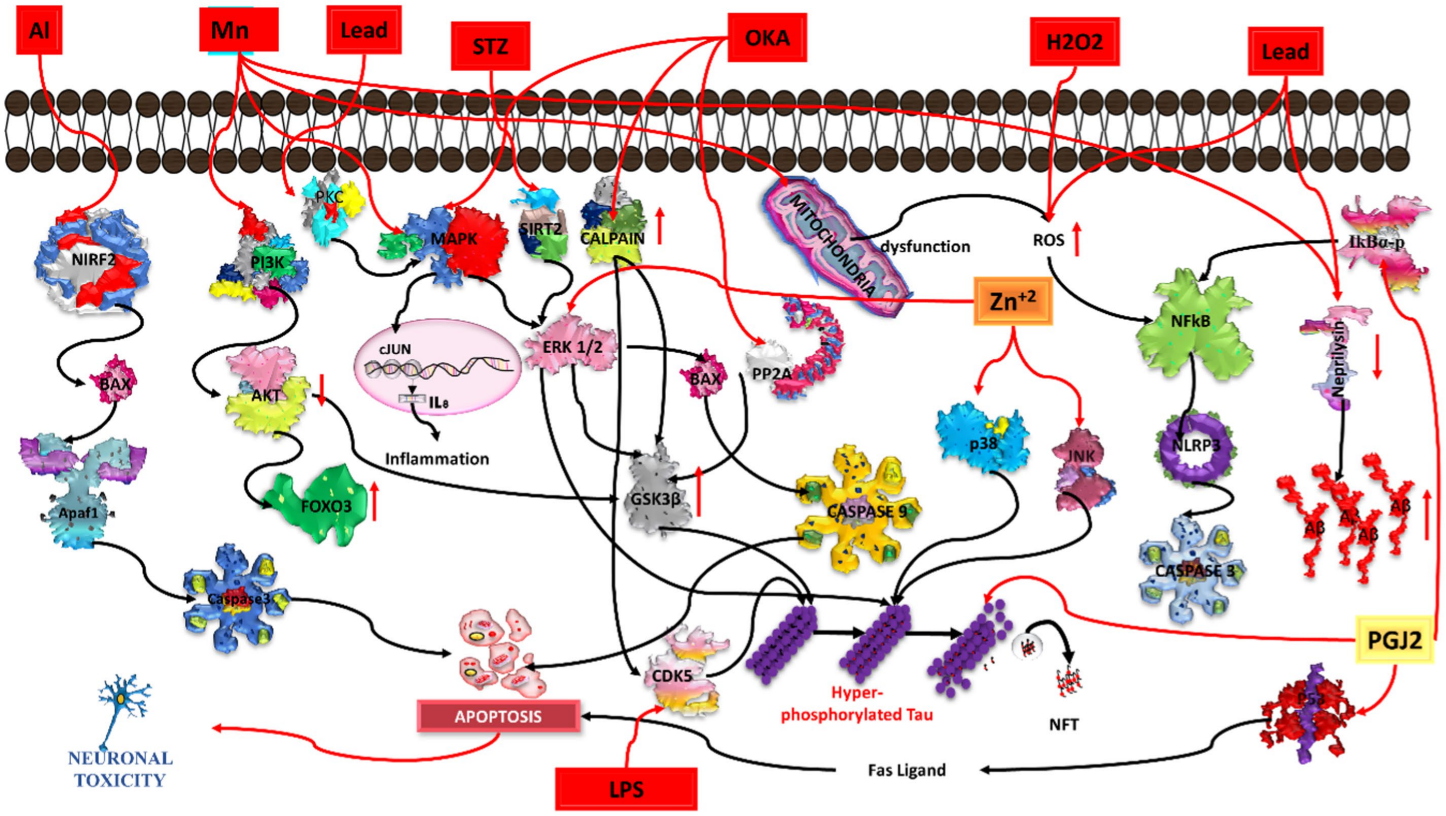
Illustration of various chemicals inducing Alzheimer’s pathology in *in-vitro* conditions. Aluminium (Al) triggers oxidative stress and neuroinflammation, contributing to beta-amyloid plaques and tau tangles. Manganese (Mn) induces mitochondrial dysfunction and neuronal damage. Lead (Pb) disrupts calcium homeostasis, promoting beta-amyloid aggregation. Streptozocin (STZ) impairs glucose metabolism, exacerbating tau pathology. Okadaic acid (OKA) activates protein phosphatases, causing hyperphosphorylation of tau. Hydrogen peroxide (H2O2) generates oxidative stress, leading to beta-amyloid accumulation. Lipopolysaccharide (LPS) induces neuroinflammation, contributing to beta-amyloid deposition and synaptic dysfunction. Common pathways include oxidative stress, neuroinflammation, and protein aggregation, while each chemical initiates specific pathways leading to neurodegeneration.

Previous studies have shown that aluminum reacts with maltolate, which is extensively present in normal human food items (baked cereals, bread, browned food, cakes, chocolate milk, and coffee), leading to the formation of aluminum maltolate Al (mal)_3_ in the intestine, which further increases the Al level in the brain and subsequently induces oxidative stress and mitochondrial dysfunction (Mashoque et al., 2018; Reitz et al., 2020; Bhatti et al., 2022). Al (mal)_3_ is further involved in transcriptional disruption rather than translational modification (Mustafa Rizvi et al., 2014). Through many *in-vitro* studies, it was found that the chronic exposure of cells to 50–100 mM AlCl_3_ caused a disruption in synapse development and promoted tau protein build-up. According to a previous study, Al (mal)_3_ and Al (acac)_3_ (aluminium acetylacetonate) can easily penetrate cells, thereby increasing the cellular toxicity. Axonal transport in cultured rat cortical neurons was found to be affected after 1-h pulse exposure to 250 mM Al (mal)_3_ (Kawahara et al., 2001). Another *in vitro* study found that Al (≤ 50 μM) enhanced the survival of cerebellar granule neuronal cells; however, higher levels of Al (≥ 100 μM) induced cell death (Tuneva et al., 2006; Figure 4).

#### 4.3.2 Manganese (Mn)

Increased exposure to Mn can cause cognitive impairment, which leads to Alzheimer disease. Mn enters the brain via diet, soil, and water, inducing apoptosis and inflammation and leading to neuronal degeneration. A higher concentration of Mn can activate MAPK/ERK/GSK3 beta signaling for hyperphosphorylation of the tau protein (Cai et al., 2011). Mn can also produce reactive oxygen species (ROS) through mitochondrial dysfunction, which further follow the activation of the NfKb/NLRP3/caspase1 signaling pathway and results in neuronal apoptosis. FOXO3 is a transcript factor expressed in the brain. It has a role in maintaining protein homeostasis; however, it is also implicated in neurodegenerative disease. A previous study suggested that Mn-exposed cells markedly increased the phosphorylation of MAPK/ERK, which further phosphorylated FOXO3; it is not able to enter the nucleus and could not transcribe the genes involved in antioxidant activity, DNA repair, and anti-apoptotic activities (Exil et al., 2014). A previous *in vitro* study found that the viability of N2a cells gradually decreases when exposed to Mn at concentrations of 50, 100, 200, 400, 800, 1,000, and 1,200 μM for 24 h. Mn also inhibited the processing of APP (amyloid precursor protein) and produced amyloid beta aggregations; however, it did not show any effect on BACE1 (beta-secretase1)–based APP processing or amyloid beta trafficking. Mn was found to decrease the expression of the synaptic protein and decrease neprilysin expression (Tong et al., 2014; Yang et al., 2021; Figure 4).

#### 4.3.3 Lead (Pb)

Lead may induce amyloid beta accumulation in differentiated SHSY5Y cells. According to a previous study, when differentiated SHSY5Y cells were treated with different concentrations (ranging between 0–5,000 μM) of lead for 24 to 72 h, no significant toxicity was observed for the lower concentrations (1, 5, 10, 20, or 50 μM) at 48 and 72 h. However, higher concentrations of lead (above 1,000 μM) reduced the cell viability at 72 h. Treatment with Pb also induces morphological alterations in neurons, thereby leading to cell damage. Higher exposure of Pb may increase AβPP (amyloid β-protein precursor) gene expression, producing more AβPP protein and further increasing the amyloid beta levels. Moreover, Pb decreases NEP (neutral endopeptidase) gene expression, reducing the production of NEP protein. The NEP protein acts as a degradation enzyme for amyloid beta. Hence, higher exposure to lead can induce increased amyloid beta levels, thereby playing a vital role in the onset of AD (Huang et al., 2011; Figure 4).

#### 4.3.4 Arsenic

**Figure 5:**
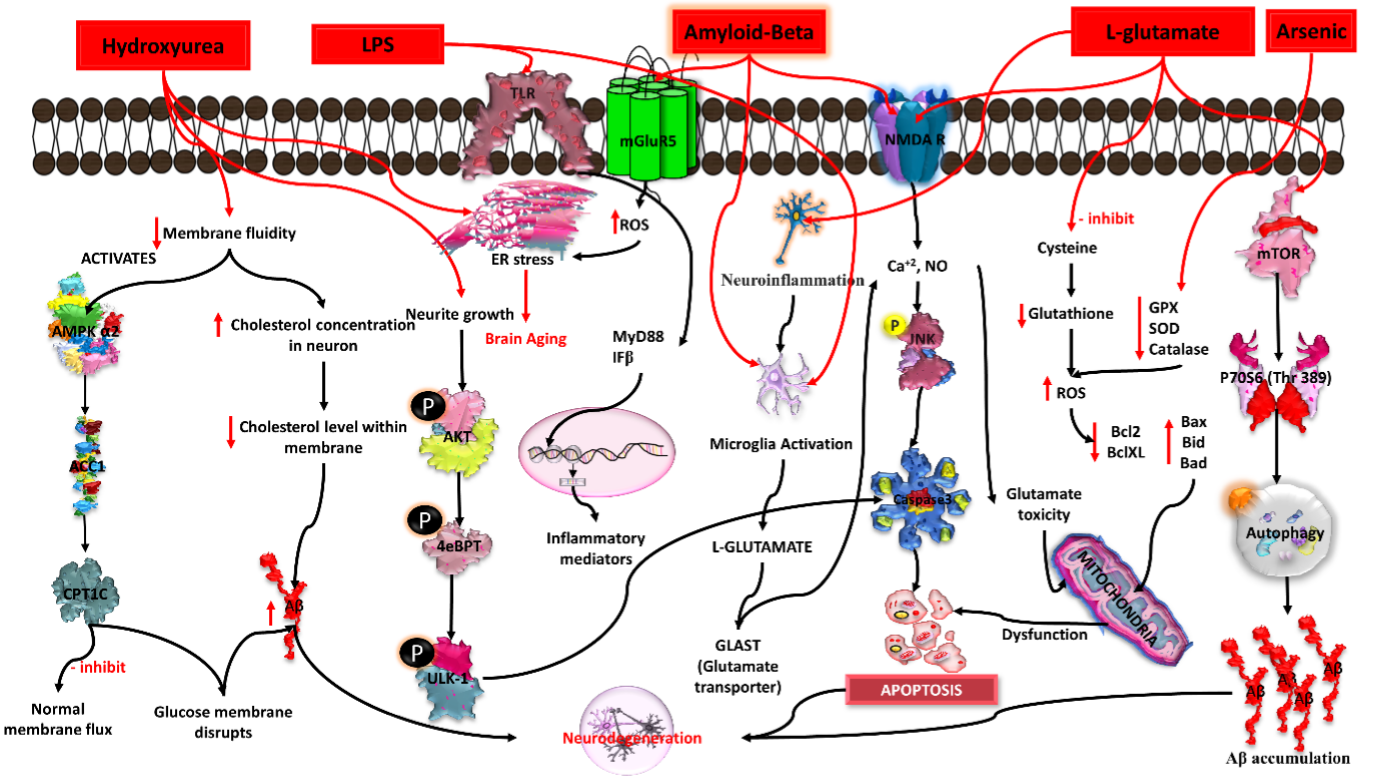
Mechanistic pathways of Alzheimer’s induction by chemical agents in *in-vitro* conditions. Hydroxyurea triggers DNA damage, lipopolysaccharide (LPS) induces neuroinflammation, amyloid-beta (Aβ) promotes plaque formation, L-glutamate contributes to excitotoxicity, and arsenic disrupts cellular homeostasis. Each agent elicits specific pathways leading to Alzheimer’s pathology, collectively portraying a comprehensive understanding of the diverse molecular mechanisms involved in disease onset.

Arsenic is a metalloid and an environmental risk factor for AD. Various studies have highlighted the role of arsenic and its derivatives in the induction and progression of AD pathogenesis. In previous studies, sodium arsenite exposure to differentiated SHSY5Y cells at a low concentration range (i.e., 1 to 10 μM) was able to decrease tau1 expression via dephosphorylation at 189–207 residues and phosphorylation at 202 residues. Furthermore, the study observed that arsenic increases GSK3 beta kinase activity and also increases the phosphorylation of ERK1/2 in neurons, thereby inducing hyperphosphorylation of the tau protein and subsequently leading to neurotoxicity as well as apoptosis (Wisessaowapak et al., 2021). Another study reported that arsenite induces neurotoxicity in the transfected Hela-p35 cell line by increasing the calcium level and activating the calpain protein (calcium dependent proteases). Calcium-induced activated calpain causes cleavage of p35 to p25, resulting in the formation of a hyper-activated complex of CDK5.p25. This complex was found to be responsible for hyperphosphorylation of tau and NFT formation (Vahidnia et al., 2008). Thus, higher exposure to arsenic upregulates the hyperphosphorylated tau protein, a hallmark feature of AD pathogenesis. A study by Rehman et al. observed that exposure of PC12 cells to arsenic (10 μM) causes neurotoxicity by increasing ROS production, leading to mitochondrial dysfunction, thereby increasing cytochrome C release and activating caspase3. Therefore, activated caspase3 causes apoptosis of neuronal cells. Additionally, arsenic also altered the protein expression of apoptosis and autophagy markers such as the downregulation of Nrf2, Bcl2/Bclx, mTOR, and Akt and the upregulation of P38 MAPK, JNK, LC3, and Bax (Rahaman et al., 2020). Hence, all these factors contribute to neurotoxicity, neuroinflammation, and neuronal loss and, ultimately, the progression of AD pathogenesis (Figure 5).

#### 4.3.5 Mercury chloride

**Figure 6:**
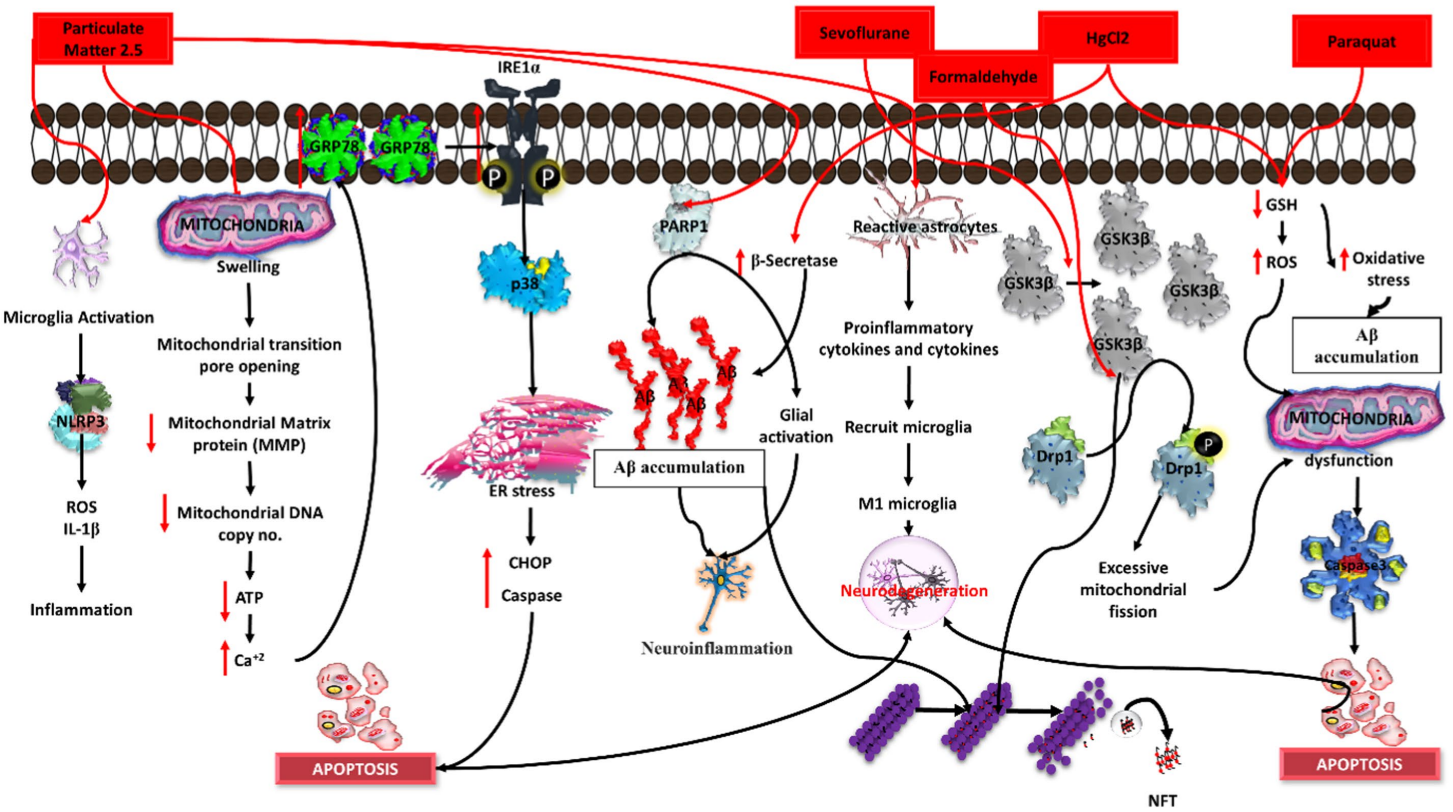
Induction of Alzheimer’s disease (AD) by distinct chemical agents in an *in-vitro* context. Particulate matter 2.5 initiates neuroinflammation, sevoflurane disrupts synaptic function, formaldehyde promotes tau hyperphosphorylation, magnesium chloride (MgCl2) induces oxidative stress, and paraquat triggers mitochondrial dysfunction. Each agent instigates specific pathways, collectively illuminating the intricate mechanisms underlying AD pathology in response to environmental and chemical exposures.

Mercury is able to induce glutamate-mediated excitotoxicity, which further leads to neuronal degeneration. Many previous studies have revealed that mercury affects glutamatergic signaling by reducing the re-uptake of glutamate, enhancing spontaneous glutamate release from neurons, thereby inhibiting glutamine synthetase, which subsequently enhances the glutamate level in the synaptic cleft. Moreover, the postsynaptic NMDA (N-methyl-D-aspartic acid or N-methyl-D-aspartate) receptor also has also been found to be affected by MeHg (methylmercury). When cortical neuronal cultures were exposed to HgCl_2_ at different concentrations, i.e., 25 nM,100 nM, and 25 μM for 3 days, neurite growth is inhibited, leading to neuronal degeneration (Xu et al., 2012). Evidence suggests that HgCl_2_ (mercury chloride) can induce oxidative stress and increase amyloid beta production, leading to hyperphosphorylation of the tau protein via that induces cellular toxicity, with reduced cell viability (Olivieri et al., 2000; Song and Choi, 2013). Mercury is also used for the development of a BBB (blood brain barrier*) in vitro* model (Toimela et al., 2004). Additionally, mercury chloride has the ability to increase β-secretase levels, which further elevate the expression of beta secretase-1 (BACE 1) and neprilysin (NEP) proteins, leading to high levels of APP (Figure 6).

#### 4.3.6 Zinc (Zn)

Zinc may regulate various pathways such as MAPK (mitogen-activated protein kinase), PI3K (phosphoinositide 3-kinase), and rapamycin-dependent pathways. When the SHSY5Y neuroblastoma cell line was exposed to 100 μM zinc sulphate, it influenced the ribosomal protein kinase and phosphorylation status of GSK3 beta. The long-term exposure of SHSY5Y cells to zinc can induce phosphorylation of various proteins such as p-PKB, p-ERK1/2, p-JNK, and p-p38, which can further alter the tau protein (An et al., 2005). Another study showed that SN56 neuroblastoma cholinergic cells uptake more zinc ions, which leads to decreased acetyl-CoA levels, ATP, and acetylcholine synthesis (Zyśk et al., 2018). Moreover, nanoparticles of zinc oxide are known to be involved in the induction of oxidative stress and inflammation in different cell types. Zinc oxide can induce apoptosis through the PI3K-AKT-caspase3/7 pathway and necrosis via LOX-mediated ROS level elevation (Kim et al., 2015; Figure 4).

#### 4.3.7 Particulate matter

Currently, research is focused on the association between AD and air pollutants, as well as the development of *in vitro* and *in vivo* study models using air pollutants. It was found that long-term exposure to certain air pollutants can induce inflammation and neuropathology, which pave the way for AD development (Wang B. R. et al., 2018). Various air pollutant particulate matter have severe health threats. Particulate matter (PM) is usually categorized into three types: ultra-fine PM (less than 0.1 μM), fine PM (less than 2.5 μM), and coarse PM (PM10 or less than 10 μM). Among these three types, PM2.5 is composed of different inorganic and organic matter, such as carbon, ammonium, hydrogen ions, metals, water, sulphates, and lipopolysaccharides (Wang B. R. et al., 2018; Figure 6).

Exposure to PM2.5 causes amyloid beta–induced neuronal injury, microglial inflammation, and an increase in the production of IL-1β and ROS, triggering cytotoxicity (Wang B. R. et al., 2018). In a previous study, PM2.5 led to apoptosis in the neuroblastoma cell line by inducing endoplasmic reticulum (ER) stress due to the overexpression of the ER molecule chaperone GRP78. Exposure to PM2.5 leads to a significant increase in IRE1α, activating p38MAPK, which promotes apoptosis by increasing the expression of the ER stress-related protein CHOP, caspase, and GRP78, further accelerating the mechanism of the neurological disease (Wang et al., 2019; Zhang M. et al., 2022). It was validated through cell culture experiment that PM2.5 was able to cross the BBB (Kang et al., 2021). PM2.5 exposure to human bronchial epithelial (HBE) cells was able to decrease SIRT1 (sirtuin1) expression. SIRT1 has a role in deacetylase activity, which further decreases tau accumulation (Lai et al., 2019). The treatment of PM2.5 in a human neuroblastoma cell line activates poly (ADP-ribose) polymerase (PARP-1), which causes Aβ accumulation and glial activation, resulting in neuroinflammation (Jang et al., 2018). The PMs evaluated in J774A.1 proliferative cells were shown to exert a cytotoxic effect after 72 h at different concentrations. Furthermore, PMs were also able to induce IL-6 and TNF-alpha secretion in J774A.1 cells (Osornio-Vargas et al., 2003). Another *in vitro* investigation found that when neuroblastoma cells (N2a-APP/Swe) were exposed to PM, their APP processing increased. Short-term PM exposure to neuronal N2a-APP/Swe cells promotes pro-amyloidogenic APP processing, as measured by the increased sAPP/ratio and Aβ42 production (Cacciottolo et al., 2017).

Exposure of human SHSY5Y cells to different concentrations of particulate matter (25, 100, and 250 μg/mL) for 24 h (Zhang M. et al., 2022) influenced the morphology of the cells, increased the mitochondrial permeability, reduced the ATP production, decreased the mtDNA replication, and increased the expression of mitochondrial genes such as Drp1 (dynamin-related protein 1), OPA1 (optic atrophy-1), CypD (cyclophilin D), COX IV (cyclooxygenase IV), and SIRT3, thereby increasing the calcium levels. This cascade of events eventually leads to apoptosis by upregulating the apoptotic pathway (CHOP/caspase-12/caspase-8) in response to an increase in oxidative stress, which causes endoplasmic reticulum stress, neurotoxicity, and neuroinflammation (Lee et al., 2021; You et al., 2022).

Therefore, PM2.5 acts as an environmental inducer of Alzheimer’s disease by penetrating the BBB and disrupting its integrity, resulting in an increase in pro-inflammatory mediators (IL-1, IL-6, and TNF-alpha), ROS, amyloid-beta production, astrogliosis, microglial activation, mitochondrial dysfunction, and ER stress. All these factors contribute to neuroinflammation and neuronal injury (synaptic impairment, tau accumulation, and neuronal death), which lead to neurological diseases such as Alzheimer’s (Kawahara et al., 2001; Huang et al., 2011; Rai et al., 2020b; Figure 6).

#### 4.3.8 Hydroxyurea

Recent *in vitro* and *in vivo* studies show that hydroxyurea-induced “membrane aging” may be exploited to create an Alzheimer’s disease model. Hydroxyurea is primarily used to treat sickle cell disease and functions as an antineoplastic agent because it decreases deoxyribonucleotides and inhibits the ribonucleotide reductase enzyme. Hydroxyurea can induce breakage near the replication sites of dsDNA and can also break the mt-DNA, leading to mitochondrial dysfunction and stress conditions and subsequently leading to senescence-like changes in cells lines (Yu and Cheng, 2021). Previous studies revealed that hydroxyurea induced Alzheimer’s disease by decreasing the membrane fluidity via the AMP-activated protein kinase (AMPK) pathway. It activates AMPK 2 and reduces the expression of its downregulating genes, acetyl CoA carboxylase (ACC1), and carnitine palmitoyl transferase 1 (CPT1C), which inhibit normal membrane flux and disturb glucose metabolism. Normal membrane flux inhibition disrupts cytoskeleton anchoring, cellular transport, intracellular communication, and neuronal transmission. Because of the disturbance in glucose metabolism, there is an increase in amyloid-beta, which leads to neuronal degeneration. Because membrane fluidity is involved in the maintenance of cholesterol levels in neurons, studies have shown that a decrease in membrane fluidity causes an increase in neuronal cholesterol levels, which decrease cholesterol levels within membrane fractions and thus cause an increase in amyloid-beta (Vahidnia et al., 2008). Induced pluripotent stem cells (iPSCs), when treated with hydroxyurea at a concentration of 1 mM, 8 mM, and 16 mM, show ER stress, a decrease in neurite outgrowth, a decrease in the expression of p-AKT, p-4EBP1, and p-ULK-1, and an increase in the expression of caspase-3, leading to apoptosis and finally causing death of neurons (Rahaman et al., 2020; Figure 5).

#### 4.3.9 27-hydroxycholesterol

**Figure 7:**
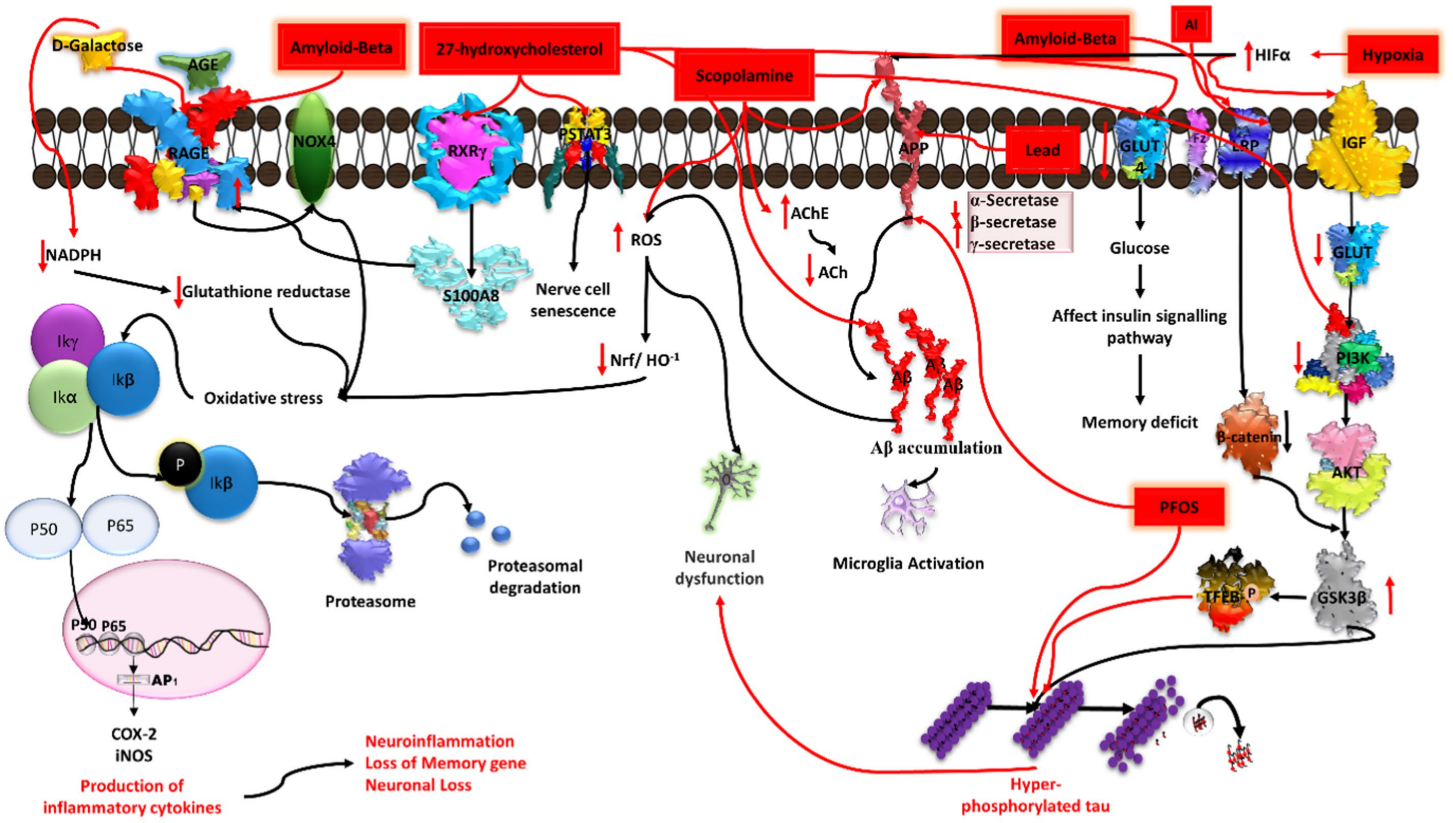
Illustration of the induction of Alzheimer’s disease (AD) by diverse chemical agents in an *in-vitro* setting. Amyloid-beta contributes to plaque formation, 27-hydroxycholesterol influences cholesterol metabolism, scopolamine disrupts cholinergic signaling, aluminium (Al) and lead (Pb) trigger neurotoxicity and oxidative stress, while hypoxia induces cellular oxygen deprivation. Each chemical agent initiates specific pathways, collectively providing insights into the multifaceted mechanisms underlying AD pathology.

27-hydroxycholesterol (27-OHC) has a role in decreasing glucose metabolism in neurons. A previous study explained that administration of 1 μM 27-OHC to rat primary neurons for 24 h leads to dampening of glucose uptake. It affects the insulin signaling pathway by decreasing GLUT-4 and increasing IRAP (interleukin-1 receptor antagonist protein) mRNA expression in cortical and hippocampal primary neurons. Because of the decrease in GLUT-4, there is a decrease in glucose uptake by neurons, which causes an imbalance in the glucose-insulin signaling pathway and leads to spatial memory deficits, an early clinical sign of AD (Ismail et al., 2017). Higher concentrations of 27-OHC were found to affect the RAGE receptor by mediating the nuclear receptor RXRγ. It influences RAGE by increasing the levels of S100A8, a calcium binding protein that acts as a ligand for the RAGE receptor. The increased level of RAGE in neurons, astrocytes, microglia, and endothelial cells leads to an inflammatory response that causes synaptic dysfunction and neurodegeneration. 27-OHC has the ability to cause oxidative stress, neuronal malfunction, and short-term memory impairment (Loera-Valencia et al., 2021). 27-OHC causes damage to astrocytic neuronal cells by activating the TGFβ/NFkB pathway, resulting in the subsequent release of inflammatory cytokines (Wang et al., 2020). Another study has shown that SHSY5Y cells treated with 27-OHC at a concentration of 5 μM, 10 μM, and 20 μM have increased levels of NFkBp65, NFkBp50, and TGF-beta and decreased expression of COX-2 (Ma et al., 2019) as well as elevated amyloid beta levels (Wang et al., 2020). Furthermore, it was found that STAT3 phosphorylated at the Tyr705 position after treatment with 27-OHC induces nerve cell senescence (Liu et al., 2021; Figure 7).

#### 4.3.10 Hydrogen peroxide

At higher concentrations, H_2_O_2_, a significant source of reactive oxygen species (ROS), can lead to oxidative stress in various cell lines. This oxidative stress can trigger apoptosis and produce alterations in signaling pathways. Previous studies have reported that H_2_O_2_ can influence amyloid precursor protein (APP) expression and gamma secretase via JNK signaling, leading to an increase in amyloid-beta (Aβ) production by reducing the protein levels of both APP and C99. Therefore, under pathological conditions, JNK may serve as a key mediator, playing a central role in facilitating the stimulatory effect of various stress signals on γ-secretase activity (Shen et al., 2008). SK-N-SH cells treated with H_2_O_2_ for 24 h showed a dose-dependent cytotoxic effect, an increase in oxidative stress, activated caspase 3 activity, and acetylcholinesterase (AChE) inhibition activity (Sattayasai et al., 2013). In another study, the administration of 100 μm of H_2_O_2_ to PC12 cells for 2 h suppressed the proliferation of cells and promoted shrinkage in the cell body, the release of LDH, increases in intra-cellular ROS, extra cellular NO, loss of MMP, which further induce apoptosis and damage dendritic networking, thereby inducing cell death (Xu et al., 2016; Zhou et al., 2018; Figure 4).

#### 4.3.11 Hypoxia

The role of chronic hypoxia/deprivation of vascular oxygen in the induction of AD pathogenesis is quite well explored, whereas low or intermittent hypoxia has a neuroprotective role. Previous studies have documented that hypoxia resulting from vascular insufficiency can increase the expression of APP. A study by Sun et al. showed the effect of hypoxia (2% O_2_ and 5% CO_2_) on SH-SY5Y cells, N2A, and HEK293 cells. The authors observed that a hypoxia-like condition activates HIF-1α, which further modulates the amyloidogenic processing of APP via increasing BACE1/α-secretase and simultaneously decreasing the β-secretase activity, ultimately leading to the formation of Aβ oligomers/aggregates. Aβ oligomers/aggregates have been found to be associated with microglia activation and thus cause neuronal cell death and inflammation (Sun et al., 2006; Lall et al., 2019).

HIF-1α also increases the ROS levels, which further leads to activation of NFκBp65, resulting in an increase in pro-inflammatory mediators. From previous studies, it has been reported that a chronic hypoxic condition also alters GLUTs and decreases ATP, which alter the insulin signaling pathway as well as PI3K/Akt/GSK3β AXIS. Activated GSK3β increases the hyperphosphorylation of tau, ultimately resulting in subsequent neuron damage/apoptosis (Lall et al., 2019). Hypoxia-induced activated HIF-1α also decreases neprilysin (a metalloendopeptidase protein) expression, leading to Aβ accumulation. Additionally, it also causes calcium dysfunction via increasing intracellular calcium concentration, subsequently leading to neuronal excitability and loss of synaptic plasticity (Zhang et al., 2007; Figure 7).

### 4.4 Chemical inducing agents

Amyloid beta, streptozotocin, L-glutamate, scopolamine, D-galactose, sevoflurane, okadaic acid, forskolin (FSK), lipopolysaccharides (LPS), PGJ2 (prostaglandin J2), and formaldehyde.

#### 4.4.1 Amyloid beta

Amyloid beta deposition occurs in the extra cellular region of neurons, further leading to AD (Stancu et al., 2014; Ramakrishna et al., 2023). Many previous studies have validated that direct injection of amyloid beta may induce alterations in primary hippocampal cortical neurons. Amyloid beta peptides of different sizes (Aβ1-40, 1.43, and 25–35) at varying concentrations are used to develop *in vitro* models of AD. Various experimental studies have highlighted the use of either synthetic amyloid beta or naturally extracted amyloid-beta peptide fragments directly from the brain of patients with AD (Jin et al., 2011). In the *in-vitro* model, amyloid beta alters tau conformation and increases tau phosphorylation as well as GSK3 beta activation (Cacciottolo et al., 2017). SHSY5Y cells exposed to an amyloid beta oligomer at concentrations of 0.01 nM to 1 nM showed decreased neurite density, morphological alteration, increased hyperphosphorylation of tau, activation of apoptotic pathway, and loss of cell viability (de Medeiros et al., 2019). Another study unveiled that amniotic membrane (AM) neuronal cells treated with amyloid beta 42 fragments (2.5 μM) for 24 h induce an AD model. The authors found a detrimental impact on the Wnt signaling pathway, impeding the neurogenesis process and triggering the activation of Gsk3 beta. This activation is involved in the hyperphosphorylation of the tau protein (Batista et al., 2019; Prajapat et al., 2020; Patil et al., 2023). Amyloid beta 1–40 and amyloid beta 1–42 at varying concentrations of 0.5 to 20 uM were able to induce apoptotic activity in PC12 cells for 24 h (Kumaran et al., 2018; Wang H. et al., 2018). Islet amyloid polypeptide (IAPP), another agent produced by pancreatic beta cells, has the ability to cross the BBB and interact with tau fibrils, thereby promoting amyloid beta production (Zhang G. et al., 2022; Figure 7).

#### 4.4.2 Streptozotocin

Streptozotocin (STZ) is a glucosamine-nitrosourea compound used as an antibiotic. An *in-vitro* model of Alzheimer’s disease has been developed using streptozotocin in various studies. Streptozotocin can modulate the activity of GSK3 alpha/beta (Bagaméry et al., 2021) and secretase, as observed in both *in vitro* and *in vivo* studies. Wang et al. found that insulin is able to inhibit betaA4 production by modulating amyloid precursor protein processing via an increase in the activity of alpha secretase and a simultaneous decrease in beta secretase activity in the APP overexpressing mutant SHSY5Y cell line. STZ was shown to exert a toxic effect in a dose dependent manner. SHSY5Y neuroblastoma cells exposed to STZ (concentration: 0.3, 1, 3, 5, and 10 mM) caused neuronal cell damage, increased oxidative stress, depolarized the mitochondrial membrane and apoptosis, and caused tau hyperphosphorylation, thereby influencing the insulin receptor expression (Bagaméry et al., 2020). A concentration of 1 mM of STZ was able to reduce the ATP concentration without cell death at 24 and 48 h (Plaschke and Kopitz, 2015). Moreover, STZ has shown toxicity in immature neuronal cells (Isaev et al., 2018). Streptozotocin inhibits the action of insulin, thereby increasing beta secretase and decreasing alpha secretase, which ultimately leads to increased amyloid beta fragments (Plaschke and Kopitz, 2015). STZ is also used to induce beta cell toxicity in the pancreas. The STZ drug is validated to generate early pathophysiological changes in cell cultures and animal AD models. STZ injected into an animal through the intraperitoneal and intracerebroventricular routes was able to reduce the cognitive behavior and increase the aggregation and deposition of amyloid beta oligomers, as well as increase the total tau protein and change the phosphorylation/total tau level ratio. Other alterations, such as a neuroinflammation, oxidative stress, and biochemical changes, have also been observed in the brain (Kamat, 2015; Sun et al., 2018; Bagaméry et al., 2020; Bansal et al., 2021; Figure 4).

#### 4.4.3 Okadaic acid (OKA)

Okadaic acid (OKA) is a polyether toxin produced by algae (Kamat et al., 2013). The main function of OKA is to inhibit PP2A (protein phosphatase 2A) and PP1A (protein phosphatase 1A) activity, and further activation of GSK3 beta, CDK5, and calpain leads to induction of abnormal hyperphosphorylation of the tau protein at positions ser202 and ser396 (Boban et al., 2019), causing tau tangle formation. Inhibition of this phosphatase activity and activation of calpain have been implicated in neurodegenerative diseases such as Alzheimer’s disease. OKA is able to activate MAPK signaling, which further activates apoptotic signaling of ERK/BAD/CASPASE9 for neuronal damage. OKA regulates various other neurotoxicity mechanisms, as observed in both *in vitro* and *in vivo* studies (Xu et al., 2004; Kamat et al., 2013). Previous *in vitro* and *in vivo* studies have shown that OKA is an oxidative stress inducer (Sachdeva and Chopra, 2015). A previous study found that exposure of primary cortical culture neurons to 50 μM of OKA and 50 μM of glutamate induces cell death (Yi et al., 2009). Further, okadaic acid was able to induce tau hyperphosphorylation, causing tangle formation, which ultimately resulted in amyloid beta deposition; therefore, it can be used to induce an *in vitro* Alzheimer’s disease model. Prior studies have shown that PC12 cells treated with OA for 24 h showed decreased cell viability, increased tau hyperphosphorylation, increased amyloid beta 1–42, and beta secretase, as well as affecting the expression of P-GSK3 beta, P-AKT, and MEF2D (Huang et al., 2020; Amonruttanapun et al., 2022; Figure 4).

#### 4.4.4 PGJ2

Prostaglandins (PG) such as PGJ2 play a key role in the inactivation of GSK3 beta by phosphorylation at the ser9 position. Numerous studies have shown that PGJ2 was able to induce hyperphosphorylation of tau in mouse cortical primary neurons. PGJ2 inactivates NFKB activity via stabilization of IKBα. PGJ2 phosphorylates IKBα for stabilization rather than degradation. Inactivated NFkB was not able to activate antiapoptotic genes or neuronal survival genes, therefore leading to neuronal cell death (Li et al., 2004). As reported in a previous study, PGJ2-treated SHSY5Y cells were responsible for the activation of the P53-dependent Fas/FasL pathway (Kondo et al., 2002).

When cells were treated with PGJ2 at concentrations of 10 μM and 20 μM, tau proteins with hyperphosphorylation at ser396, ser404, and thr205 were found to be upregulated (Mpousis et al., 2016; Thysiadis et al., 2018). PGJ2 acts as an inducing model for AD. On the other hand, AcPHF6 (acetyl PHF6) derivatives are able to induce fibrillation of cross beta sheet structures and tau aggregation in cell cultures. Therefore, it can be a suitable agent for the induction of an *in vitro* model for Alzheimer’s disease (Lunven et al., 2016; Figure 4).

#### 4.4.5 Scopolamine

Scopolamine is a muscarinic acetylcholine receptor antagonist that induces cognitive impairment by increasing oxidative stress, inflammation, and the inhibition of cholinergic neurotransmission. Exposure of human neuroblastoma SHSY5Y cells to 1 to 3 mM concentration of scopolamine for 24 h was able to decrease the cell viability. In another study, it upregulated the AChE activity in rat PC12 cells (Puangmalai et al., 2017). Exposure to scopolamine decreases PI3K/Akt, which activates GSK3β, ultimately resulting in subsequent neuron damage/apoptosis via increasing the hyperphosphorylation of tau. Scopolamine exposure to IMR32 neuronal and C6 glioma cells at a dose of 3 mM for 2 h induces oxidative stress that leads to an alteration in downstream signaling pathways such as NFκB-mediated CREB/BDNF and GFAP activity, leading to neuroinflammation, apoptosis, synaptic dysfunction, and loss of memory genes (Konar et al., 2011; Figure 7).

#### 4.4.6 L-glutamate

The excitatory neurotransmitter in CNS is glutamate, which can cause neurodegeneration in animal models, primary cultures of brain cells, and cell lines such as PC12. In a study involving the PC12 cell line, a high concentration of glutamate was able to inhibit the uptake of cystine and reduced glutathione intracellularly, which lead to the induction of ROS, ultimately causing cell death (Yu et al., 2005). Studies have found that L-glutamate leads to excitotoxicity in human neuroblastoma SH-SY5Y cells when treated with different concentrations (0.1–10 mM) for 12 h. The overactivation of NMDA receptors induces glutamate excitotoxicity due to the large influx of Ca^+2^ and NO. This causes activation of the JNK signaling pathway, which further activates caspase-3 and pro apoptotic genes, ultimately leading to apoptosis and neuronal cell death (Bagaméry et al., 2021). Previous research has also linked glutamate excitotoxicity to a variety of neurodegenerative diseases, retinal diseases, hyperglycemia, diabetic retinopathy, and type 3 and type 4 diabetes (Wang et al., 2019). A high concentration of L-glutamate not only causes excitotoxicity but also affects the tau protein in rat primary culture neurons (Chen et al., 1995). It has been suggested that there is involvement of MAPK, PI3K/Akt, and mTOR signaling pathways in L-glutamate-induced excitotoxicity (Plaschke and Kopitz, 2015). Microglial cells were also found to release a high concentration of glutamate, which decreases the level of glutamate transporters (GLAST1) and results in glutamate-induced excitotoxicity (Takaki et al., 2012; Figure 5).

#### 4.4.7 Galactose-D

Mild Alzheimer’s and dementia can also be induced by D-galactose, which is a reducing sugar naturally occurring in our body and present in various food products. However, an excess amount of D-galactose can induce oxidative stress, apoptosis, and inflammation in neuronal cells. Previously, it was reported that D-galactose can induce senescence and further neuronal damage in both *in vivo* and *in vitro* AD models. One such study highlighted that human SHSY5Y cells exposed to D-galactose at a concentration of 300 mM for 48 h exhibited enhanced inflammation, apoptosis, oxidative stress, and mitochondrial dysfunction since D-galactose is used to mimic the natural aging process.

Other studies by Wang et al. and Rahimi et al. suggested that exposure of 200 mM to 400 mM D-galactose to retinoic acid induced differentiated SHSY5Y cells that can cause senescence and oxidative stress by decreasing NADPH glutathione reductase (Lee et al., 2020). A high amount of D-galactose reacts with peptides and forms advanced glycation end products (AGEs) in the body, which interact with receptor of advanced glycation end products (RAGE). This RAGE is present in the membranes of various cells, such as dendritic cells, neuronal cells, macrophages lymphocytes, glial cells, smooth muscles cells, and endothelial cells. RAGE was found to cause oxidative stress and inflammation by acting on Nox4 (Rahimi et al., 2018). Astrocytic CRT cells and rat primary astrocytes treated with D-gal were found to have increased NFκBp65 and therefore increased pro-inflammatory mediators. Furthermore, cells showed decreased cell viability and pronounced occurrence of cellular senescence due to an increase in caspase3 (Hou et al., 2019; Figure 7).

#### 4.4.8 Sevoflurane

According to previous *in vivo* studies, sevoflurane can induce cognitive impairment and tau hyperphosphorylation. *In vitro* studies have shown that when a neuronal culture was exposed to sevoflurane, it increased the amount of tau and also induced phosphorylation of tau at 202 and 205 residues (Dong et al., 2021). Other studies reported that sevoflurane was able to induce caspase-3 activity, causing cytochrome C release and apoptosis. It can alter the processing of the amyloid precursor protein (APP) and can also increase the levels of amyloid beta peptide fragments, as well as elevate the intracellular BACE (beta-secretase 1) levels in the brain tissue of mice, leading to cellular damage (Wei et al., 2014). SK-N-SH cells treated with an approximate concentration of 10 mM of sevoflurane showed upregulation of GSK3 beta protein, accelerating the phosphorylation of Drp1 at ser616 residues, which further leads to damaged mitochondria and ultimately leads to apoptosis via caspase 3 activation (Boost et al., 2009; Liu et al., 2022). Sevoflurane-induced neuronal toxicity has been one of the pieces of evidence for AD pathogenesis in cell culture and animal studies (Huang et al., 2018; Figure 6).

#### 4.4.9 Formaldehyde

Formaldehyde is a common environmental agent found in various products such as paint, cloth, exhaust gas, medicinal items, and industrial goods. It is a natural by-product of metabolism in living organisms. DNA/protein crosslinks are produced when exposed to formaldehyde, suggesting a key mechanism of DNA damage. Surprisingly, these crosslinks have been utilized to calculate medicine dose. Formaldehyde also works as a crosslinking agent, interacting with thiol and amino groups to cause protein polymerization.

Furthermore, formaldehyde may interact with Aβ, resulting in the development of neurotoxic amyloid-like complexes that are permanently cross-linked. The effects of formaldehyde-induced tau aggregation on human neuroblastoma cells (SHSY-5Y cell line) and rat hippocampus cells were studied. The researchers discovered that very modest quantities of formaldehyde (0.01–0.1%) are enough to cause the production of amyloid-like tau clumps. Consequently, both SH-SY5Y and hippocampus cells undergo apoptosis as a result of these aggregates (Nie et al., 2007). According to a study by Lu et al., a higher concentration of formaldehyde can damage the central nervous system. It can cross the BBB. In this study, the authors exposed N2A cells to 0.5 mM formaldehyde for 24 h. Formaldehyde can trigger hyper phosphorylation of the tau (p396) protein through the activation of GSK3 beta, which further leads to neuronal cell death (Lu et al., 2013; Figure 6).

However, apart from environmental and chemical inducing agents, xenobiotics and xenometals can also be considered as important inducing agents for the pathogenesis of Alzheimer’s disease. Previous *in vivo* studies have demonstrated the significance of such agents in a rodent model of Alzheimer’s disease (Mahdi et al., 2019). In the context of xenobiotics and xenometals, they were shown to repress anti-aging genes such as sirtuin 1 (Martins, 2016, 2017). Sirtuin 1 plays a very important role in mitophagy, amyloid beta aggregation, and programmed cell death, and is a critically important gene for AD (Martins, 2018).

### 4.5 Other AD inducing agents

#### 4.5.1 Herbicides

Paraquat is a herbicide that was found to be involved in neurodegeneration. According to a previous study, when differentiated SHSY5Y cells were treated with paraquat, it induces downregulation of GSH levels, which in turn leads to high levels of ROS in the mitochondria. Further, paraquat also has the ability to elevate caspase 3 levels and proteasome, which leads to apoptosis and further leads to neuronal cell death (McCarthy et al., 2004; Figure 6).

#### 4.5.2 Perfluorooctanesulfonic acid (PFOS)

PFOS is an organic pollutant able to cause neuronal toxicity and is a potential risk factor for AD in late life. Exposure of SHSY5Y cells to PFOS induced tau pathology via the induction of specific phosphorylation of the protein, resulting in a high expression of GSK3 beta, thereby increasing the expression of APP and inducing high APoE levels (Basaly et al., 2021; Figure 7).

#### 4.5.3 Lipopolysaccharides

Lipopolysaccharide (LPS) is located in the outer membrane of Gram-negative bacteria, with the toll like receptor (TLR) as its main receptor, although it exerts its effects via other receptors. LPS interacts with TLR to further recruit downstream adaptors, such as MyD88, TIR-domain-containing adaptor-inducing interferon-β, and/or triggering receptors expressed on myeloid cells (TRAM). All these adaptors can activate transcription factors responsible for the activation of pro-inflammatory genes. LPS can also induce microglial astrocytic cells as both of them express the TLR4 receptor. Indeed, activation of this receptor leads to the production of different inflammatory mediators. LPS-induced inflammation exacerbates tau pathology through cdk5 kinase activity (Sun et al., 2006), resulting in amyloid beta deposition, as observed in both *in vitro* and *in vivo* studies (Bansal et al., 2021). Astrocytes are major players in the development of diverse neurodegenerative diseases. Many of the neuroinflammatory models developed *in vitro* (cultured astrocytes) and *in vivo* (mouse) were accomplished using LPS in numerous studies (Liu et al., 2019; Creative Biolabs, n.d.; Figure 5).

## 5 Discussion

The lack of ability to perform a systematic analysis on *in vitro* studies makes the extrapolation of data to a patient population much more challenging. The present study aims to identify the different compounds that were used to generate *in-vitro* models of Alzheimer’s disease due to their toxic nature toward humans—generating a model that can more closely imitate the pathological features of AD subtypes. However, various *in vivo* Alzheimer’s models have been employed to explore the molecular pathways, and testing the efficacy of drugs has also been successfully carried out. As neuronal toxicity models exhibit physiological relevance in terms of amyloid beta plaque formation and tau hyperphosphorylation, their clinical prognostic utility will be helpful in evaluating single drug effects as well as drug–drug combinations. A method to develop an *in vitro* Alzheimer’s disease model using multiple chemicals has been established to untangle AD pathogenesis, identify molecular processes, and test numerous prospective therapeutic drugs against AD. This applies especially to metal ions, particulate matter, and chemical inducers that have potential to cause Aβ plaques and neurofibril tangles. Nonetheless, none of the existing models accurately reproduce the pathology of human Alzheimer’s disease, even though each *in-vitro* model is recognized to have its own advantages.

Heavy metals, PGJ2, okadaic acid, scopolamine, amyloid beta, and LPS are among the chemicals with proven neurotoxicity and have been used to upregulate Alzheimer-specific genes in differentiated neuronal cells. However, to trigger cellular AD pathology, each chemical may work through different approaches and different signaling pathways. The aim of the present study is to identify a common molecular mechanism and the advantages of each model need to be considered while screening drugs for AD research. Each type of chemical that is being used for creating *in vitro* AD models replicates certain stages of AD pathophysiological alterations and their associated molecular mechanisms, which should be carefully considered when choosing an *in vitro* AD model.

Cerebral organoids that allow the production of ordered structures akin to the human brain have recently become the newest route for *in vitro* AD research (Kumaran et al., 2018). These latest 3D *in vitro* models might have the potential to offer new prospects toward the exploration of personalized therapeutic regimes and new strategies.

## 6 Conclusion

The development of methods for obtaining a functionally meaningful AD model will aid in increasing the predictive capacity of new therapeutic drug screening agents. However, for these models to be accepted by industry, higher-throughput screening procedures that can be assessed by an automated system would be required.

The inducible *in vitro* AD models reviewed in this comprehensive review have helped us to identify different compounds that are suitable to be used for the development of *in-vitro* models of Alzheimer’s disease capable of more closely imitating the pathological features of AD subtypes. Regardless, none of these models can effectively imitate all aspects of disease development in most AD subtypes. As a result, current models will need to be modified to adequately replicate the complex scenarios occurring during the actual manifestation of Alzheimer’s disease. Nevertheless, chemically induced AD models will continue to play an essential part in future Alzheimer’s disease research.

## Data availability statement

The original contributions presented in the study are included in the article/supplementary material, further inquiries can be directed to the corresponding author.

## Author contributions

MP: Conceptualization, Methodology, Writing – original draft. GK: Conceptualization, Data curation, Methodology, Writing – original draft. GC: Data curation, Investigation, Software, Writing – original draft. PP: Methodology, Writing – original draft. SB: Investigation, Software, Writing – review & editing. RJ: Software, Visualization, Writing – review & editing. GB: Visualization, Writing – original draft. AM: Investigation, Visualization, Writing – review & editing. RS: Writing – original draft. HK: Writing – review & editing. PKP: Writing – review & editing. AP: Supervision, Validation, Writing – review & editing. BM: Supervision, Validation, Writing – original draft.
